# Rod-like Cellulose Regenerated by Bottom-Up Assembly in Natural Rubber Latex and Its Reinforcement

**DOI:** 10.3390/ijms24076457

**Published:** 2023-03-30

**Authors:** Haoze Yuan, Peixing Li, Xinyu Wang, Hongying Zhao, Jutao Sun

**Affiliations:** 1 School of Polymer Science and Engineering, Qingdao University of Science & Technology, Qingdao 266042, China; 2 Sino-German College of Science and Technology, Qingdao University of Science & Technology, Qingdao 266042, China

**Keywords:** regenerated cellulose, bottom-up, cellulose solution, natural rubber latex

## Abstract

As a renewable biomass material, nano-cellulose has been investigated as a reinforcing filler in rubber composites but has seen little success because of its strong inclination towards aggregating. Here, a bottom-up self-assembly approach was proposed by regenerating cellulose crystals from a mixture of cellulose solution and natural rubber (NR) latex. Different co-coagulants of both cellulose solution and natural rubber latex were added to break the dissolution equilibrium and in-situ regenerate cellulose in the NR matrix. The SEM images showed that the sizes and morphologies of regenerated cellulose (RC) varied greatly with the addition of different co-coagulants. Only when a 5 wt% acetic acid aqueous solution was used, the RC particles showed an ideal rod-like structure with small sizes of about 100 nm in diameter and 1.0 μm in length. The tensile test showed that rod-like RC (RRC)-endowed NR vulcanizates with pronounced reinforcement had a drastic upturn in stress after stretching to 200% strain. The results of XRD and the Mullins effect showed that this drastic upturn in stress was mainly attributed to the formation of rigid RRC-RRC networks during stretching instead of the strain-induced crystallization of NR. This bottom-up approach provided a simple way to ensure the effective utilization of cellulosic materials in the rubber industry.

## 1. Introduction

Natural rubber (NR) is a renewable resource derived from latex found in the sap of some plants. It exhibits high elasticity and tensile strength and good biocompatibility and biodegradability; thus, it is widely used in tires, belts, gloves, and many other applications. NR compounds are usually filled with a large amount of reinforcing filler to improve their performance and reduce their cost. Carbon black and silica are the two most commonly used reinforcing fillers in the rubber industry. However, both of them have the disadvantages of high pollution, high energy consumption, high density, and non-degradability. Cellulose is the most abundant natural polymer on earth. Native cellulose includes crystalline regions and amorphous regions. By removing most of the amorphous regions, crystalline cellulose, including microcrystalline cellulose (MCC) and nanocrystalline cellulose (NCC), can be obtained. The crystalline cellulose has a high strength and modulus and a great potential for use as a reinforcing filler in rubber materials [1,2].

A “top-down” approach is usually taken to prepare MCC and NCC by directly removing amorphous regions from the native cellulose through acid hydrolysis or enzymolysis [3,4]. MCC is cheap and commercially available but is limited to only being used as a reinforcing filler because of its large size (20–90 μm) and weak bonding with the hydrophobic rubber matrix. NCC can be prepared by further acid hydrolysis of MCC or native wood. NCC is composed of rigid, rod-like particles with a width of less than 100 nm and lengths of up to hundreds of nanometers. The size and geometrical features of NCC play important roles in the mechanical properties of reinforced composites [5,6,7]. However, the nanoscale size and rod-like geometry also lead to a strong tendency to form serious aggregates during the drying process (a necessary step for the “top-down” approach) [8]. The NCC aggregates cannot be uniformly dispersed into the rubber matrix [9]. Many chemical modification methods, such as esterification [10], acylation [11,12,13], and grafting [14], have been developed to avoid aggregating during the drying process. However, chemical modifications made expensive NCC even more expensive, which seriously restricts its effective application in the rubber industry. The cellulose nanofiber (CNF) is also an important cellulose product that has been used in rubber reinforcement, but the entanglement between fibers will result in a decrease in elongation at break [15].

In recent years, it has been found that cellulose could be dissolved in an alkaline/urea aqueous system [16,17]. Regenerated cellulose (RC) can precipitate from the solution by heating it or adding certain chemicals [18,19]. The method that can get RC from a cellulose solution is called the bottom-up approach. This approach provides new insight into the application of cellulose in polymer composites. Usually, the RC particles were pre-prepared in the form of porous gel and then mixed with various monomers, such as methyl methacrylate and ε-caprolactone. The composites were obtained by the in-situ polymerization of the monomer in the porous gel. This methodology is suitable for RC/thermoplastic composites but has some limitations for use in rubber composites due to the high viscosity of rubber materials. Yu et al. [20,21] prepared RC/rubber films with an IPN (interpenetrating polymer network) or semi-IPN structure by regenerating cellulose in NR latex. The RC particles showed a honeycomb-like structure at a microscale size.

In this paper, the cellulose/alkaline-urea-aqueous solution was pre-treated to a critical state and then mixed with NR latex to form a homogenous solution. The RC particles were precipitated in an NR matrix through a co-coagulation process by adding certain coagulants. The RC/NR hybrids were then mixed with other additives, such as sulfur and an accelerator, to prepare the final vulcanizates. The goals of the present study consist of determining the conditions for in-situ regeneration of rod-like RC (RRC) particles in an NR matrix and evaluating the reinforcement effect of RRC particles on NR compounds.

## 2. Results

### 2.1. Micromorphologies of MCC and RC/NR Hybrids

**Figure 1:**
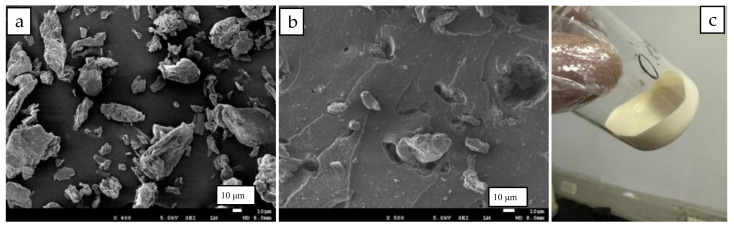
(**a**) SEM image of MCC; (**b**) SEM image of the tensile fracture of MCC/NR composites; (**c**) optical photo of cellulose solution mixed with NR latex.

Raw microcrystalline cellulose (MCC) has large sizes of 20–90 μm and irregular shapes, as shown in the SEM photo in Figure 1a. By directly mixing them with NR latex to prepare composites, the large sizes and high polarity resulted in weak interfacial bonding. It was observed from the SEM photo of the tensile fracture in Figure 1b that MCC particles had been almost completely separated from the rubber matrix, and some of them had even been pulled out. As a result, raw MCC particles were not suitable to be used as reinforcing filler in rubber materials.

MCC particles were completely dissolved in alkaline-urea-aqueous and formed a transparent cellulose solution with a few bubbles. The cellulose solution was then mixed with NR latex by simply agitating it, which then formed a uniform mixture, as shown in Figure 1c. Commercial NR latex showed strong alkalinity because it had been stabilized in advance by adding a certain amount of ammonia. The strong alkalinity of NR latex ensured its compatibility with alkaline cellulose solutions.

**Figure 2:**
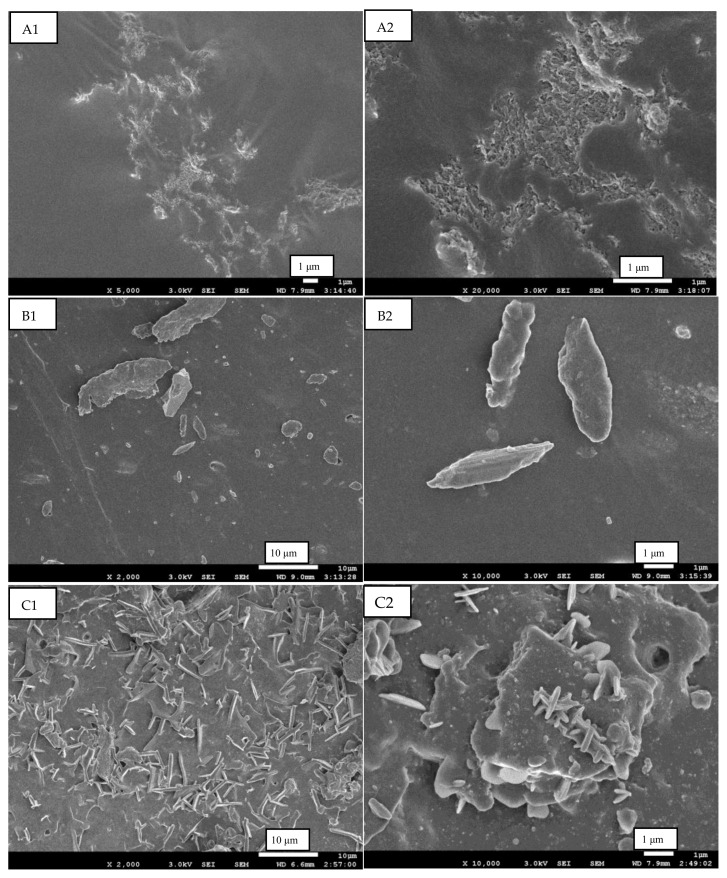
SEM images of the tensile fracture of RC/NR hybrids. (**A1**,**A2**): hybrid obtained by adding ethanol; (**B1**,**B2**): hybrid obtained by adding 5 wt% CaCl_2_ aqueous; (**C1**,**C2**): hybrid obtained by adding glacial acetic acid.

Due to the strong intermolecular and intramolecular hydrogen bonding among cellulose chains, the dissolution equilibrium of the cellulose solution is easy to break by heating, diluting, or adding certain chemical ingredients. The cellulose chains then tended to self-assemble into cellulose crystals and precipitate from the dissolved state. Based on this foundation, together with the mixture of cellulose solution and NR latex, a co-coagulation method was developed to prepare RC/NR hybrids. The “co-coagulation” meant that both cellulose crystals and demulsified NR macromolecules were formed bottom-up almost synchronously by adding certain chemical ingredients to the mixture. In principle, all chemicals that can coagulate both a cellulose solution and NR latex were possible candidates. Herein, glacial acetic acid, ethanol, and 5 wt% CaCl_2_ aqueous were chosen, respectively, as co-coagulants, and their effects on the micromorphologies of RC particles were shown in Figure 2.

The micromorphologies of RC particles in the NR matrix changed obviously by adding different co-coagulants, as observed in Figure 2. When ethanol was used as a coagulant, it showed in Figure 2(A1,A2) that the RC particles had a unique honeycomb-like structure and were dispersed at the micro-scale instead of the nano-scale. However, when 5 wt% CaCl_2_ aqueous was used as the coagulant, the RC particles in Figure 2(B1,B2) showed a short-rod structure with very large sizes, with diameters of 1–2 μm and lengths of 2–10 μm. Figure 2(C1,C2) were the morphologies of RC particles when glacial acetic acid was used as a coagulant. The fiber-like RC dispersed randomly in the NR matrix with a length of about 10 μm and a diameter of 0.1–0.4 μm. The difference in sizes and geometrical features of RC particles would result in different reinforcement effects in the NR matrix.

### 2.2. Mechanical Properties of RC/NR Vulcanizates

The RC/NR hybrids that were obtained by adding different coagulates were mixed with other curing ingredients, as listed in Table 1, to prepare the final RC/NR vulcanizates. The NR in the controlled sample was directly coagulated from NR latex instead of a mixture of NR latex and cellulose solution.

The mechanical properties, including tensile strength, tear strength, 300% modulus, and elongation at break, of different RC/NR vulcanizates were measured by a tensile test, and the results are listed in Table 2.

The tensile strength and tear strength of all RC/NR samples were slightly higher than those of the control. Especially for the sample in which glacial acetic acid was used as a coagulant, it had the highest mechanical performance and a great improvement of 300% modulus, yet this was accompanied by a decrease in elongation at break.

### 2.3. Micromorphologies of Rod-like RC Particles

**Figure 3:**
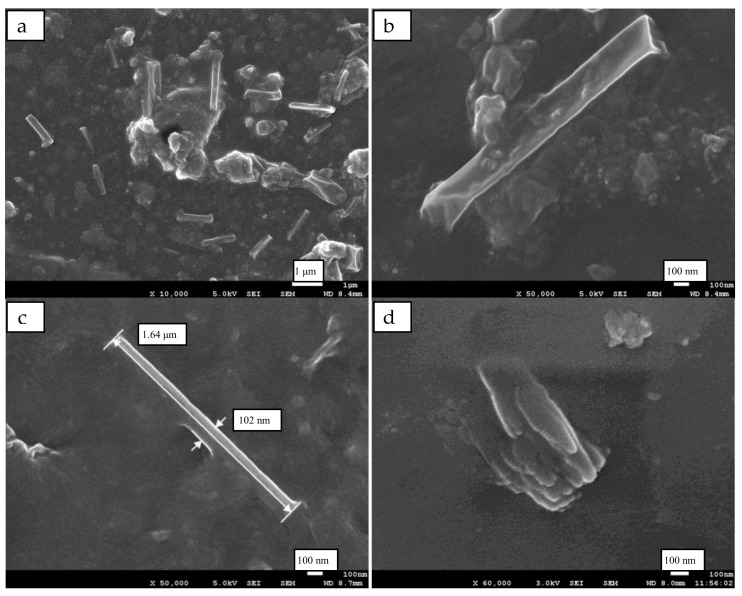
SEM images (**a**) of the tensile fracture of RRC/NR composites. (**b**,**c**) are the amplification of (**a**); (**d**) is the unseparated RRC.

To further control the sizes and shapes of RC particles, the co-precipitation rate of cellulose chains and NR macromolecular was adjusted by diluting the glacial acetic acid to a 5 wt% aqueous solution, and the micro-morphologies of RC particles in the NR matrix were shown in Figure 3. The RC particles exhibited ideal rod-like shapes and dispersed well in the NR matrix in Figure 3a. The rod-like RC (RRC) particles had a length of about 1.0 μm and a diameter of about 100 nm. Its sizes and geometry were similar to those of the NCC. The RRC particles exhibited good interfacial bonding with the rubber matrix, as shown in Figure 3b,c. Still, some aggregates of regenerated cellulose crystals appeared in Figure 3d, which indicated that completely preventing the regenerated nanocrystals from aggregating was very difficult.

### 2.4. Mechanical Properties of RRC/NR Vulcanizates

**Figure 4:**
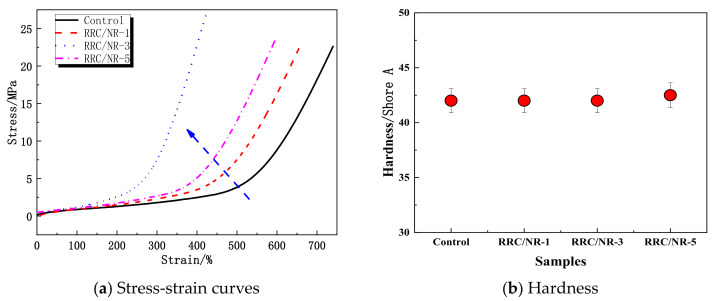
(**a**) Stress-strain curves and (**b**) hardness of the vulcanizates. The blue arrow only guide your eyeline.

Using a 5 wt% acetic acid aqueous solution as a coagulant, different loadings of RRC particles were regenerated in the NR matrix to prepare RRC/NR hybrids. The hybrids were mixed with other ingredients in Table 1 and then cured to prepare vulcanizates. The tensile properties (stress-strain curves) and hardness of RRC/NR vulcanizates were measured and compared with the control in Figure 4a,b, where RRC/NR-1, RRC/NR-3, and RRC/NR-5 represented the loading of RRC particles 1, 3, and 5 phr, respectively.

As shown in Figure 4a, the tensile strength of all RRC/NR samples was higher than that of the control and reached its maximum value when the RRC was 3 phr, increasing from 22.6 MPa to 26.8 MPa. There was a shoulder or plateau at about 500% strain for the control, and further increases in the strain led to a sharp upturn in stress. While for the RRC/NR samples, the onset of such an upturn declined and was related to the loading of RRC particles. Especially for RRC/NR-3, the onset strain was only 200%. In addition, it was notable that the loading of the RRC had almost no effect on the hardness, as shown in Figure 4b, regardless of whether the modulus changed or not.

**Figure 5:**
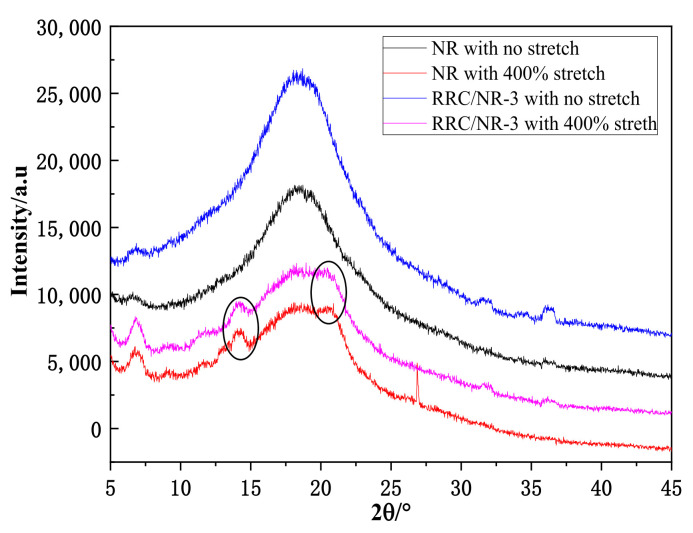
XRD patterns of the NR and RRC/NR samples. The black circle only guide your eyeline.

To determine the root cause of the declination of the onset strain, the XRD patterns and Mullins effect of the RRC/NR vulcanizates were compared with those of the control. The samples were pre-stretched to 400% strain and measured by XRD at room temperature. The XRD patterns of the control and RRC/NR-3 samples are displayed in Figure 5.

There was a broad hump located around 19° for all measured samples, which was the amorphous diffraction peak of cured NR [22]. After stretching to 400% strain, two peaks at 14.3° and 20.6° appeared for the NR sample, which were caused by the crystals of stretched NR. The diffraction peaks at 14.3° and 20.6° were attributed to the (201) and (002) crystalline planes of the monoclinic crystal [22]. These two peaks (14.3° and 20.6°) also appeared in the stretched sample of RRC/NR-3 and have almost the same intensity as the pure NR sample.

**Figure 6:**
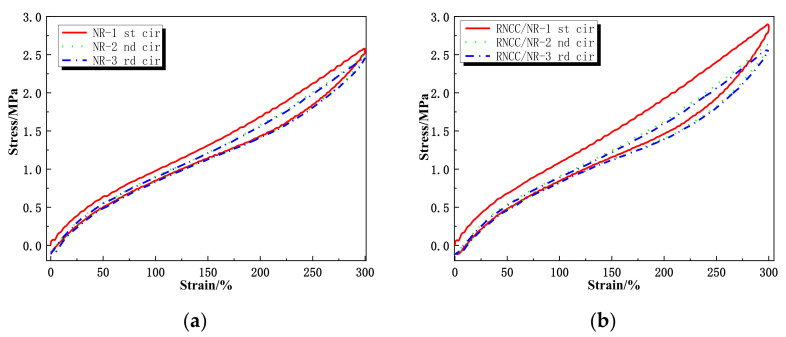
Mullins effect of the NR and RRC/NR samples. (**a**) Mullins effect of NR; (**b**) Mullins effect of RRC/NR.

Figure 6a,b show the cyclical loading and unloading of a 300% strain of NR and RRC/NR-3 vulcanizates, carried out on an electron tensile testing machine.

It was observed that the unloading and reloading paths of the stress-strain diagrams differed substantially, and the successive relaxation paths were situated each below the previous one. This was the well-known strain-softening phenomenon (or damage accumulation), usually named the Mullins effect in the rubber industry. When comparing Figure 6a with Figure 6b, the RRC/NR composites showed a much more obvious Mullins effect than that of the control under cyclical loading.

**Figure 7:**
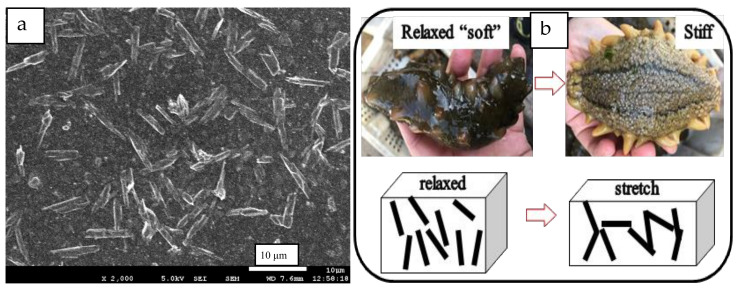
(**a**) SEM image of RRC dispersed in the NR matrix; (**b**) schematic diagram of the RRC networks formed under stretching.

Figure 7a is a SEM image of the tensile fracture of RRC/NR-3 vulcanizate. It shows that the RRC particles dispersed well in the NR matrix, but to a slightly larger degree than what is shown in Figure 3. Figure 7b is the schematic diagram of the RRC networks that formed under stretching.

## 3. Discussion

### 3.1. Micromorphologies of RC Particles and Their Reinforcement

Controlling the sizes and shapes of RC particles was a hard nut to crack when using the co-coagulation method. The precipitating rates of the cellulose chain and NR molecular were quite different when a certain coagulant was added to the mixture of cellulose solution and NR latex, which resulted in different micromorphologies of RC particles in the NR matrix.

Ethanol was an efficient coagulant for both the cellulose solution and the NR latex, which meant that both the cellulose phase and the NR phase would be rapidly and synchronously formed bottom-up when ethanol was added to the mixture. The cellulose phase precipitated so quickly that it hardly had time to self-assemble into ideal crystals and finally formed a honeycomb-like structure, Figure 2(A1,A2), due to interaction with the NR chains that precipitated simultaneously. The honeycomb-like structure of RC was considered beneficial to the mechanical properties of the composites [20], but its large size greatly deteriorates its reinforcement efficiency, as shown in Table 2.

CaCl_2_ aqueous was a commonly used demulsifier for NR latex, but the demulsification process was slow, usually needing 8–16 h. However, it was fairly efficient to break the dissolution equilibrium of the cellulose solution because of the reaction of OH^−^ with Ca^2+^. A large number of cellulose chains suddenly separated from their dissolved state and quickly self-assembled into short-rod structures with massive sizes, Figure 2(B1,B2), due to the lack of NR chain interference. Though the size of the short-rod RC particles was still very large, their aspect ratio resulted in higher mechanical properties than those of the honeycomb-like RC.

Glacial acetic acid was a moderate coagulant for both the cellulose solution and the NR latex. The regenerated cellulose chain precipitated and self-assembled into a fiber-like shape, Figure 2(C1,C2), under the interaction of the NR macromolecular chain that had precipitated simultaneously. The fiber-like RC had a high aspect ratio and thus acquired the highest reinforcement efficiency. However, the fiber-like RC was susceptible to entanglement under strain, which resulted in a 300% modulus decrease.

In a word, the fiber-like RC particles exhibited certain reinforcement efficiency in the NR matrix but were not very strong. Its sizes and shapes needed to be further controlled. By diluting glacial acetic acid to a 5 wt% solution, the precipitating and self-assembly rates of the cellulose crystals were reduced, and thus their further growth into a fiber-like state was avoided. The NR macromolecules, separated from the latex simultaneously, interacted with the precipitated cellulose crystals and prevented them from aggregating. Therefore, rod-like RRC particles with small sizes were obtained and dispersed well in the NR matrix. The small size and geometrical features of the RRC particles, together with their good dispersion and interfacial bonding, resulted in their high reinforcement efficiency in NR compounds.

### 3.2. Mechanical Properties of RRC/NR Vulcanizates

When the loading of the RRC was 3 phr, the RRC/NR vulcanizates showed satisfactory mechanical properties. Furthermore, it was interesting to note that its stress-strain curves had a sharp upturn of stress at only 200% strain, whereas the upturn was at 500% strain for pure NR vulcanizates (the control). For pure NR vulcanizates, the origin of such a sharp upturn was attributed to the well-known train-induced crystallization (SIC) of NR [23]. For the reduction in the onset strain of RRC/NR-3 vulcanizates, there were two possible reasons: one was attributed to the highly ordered architectures of the RRC particles, which made the SIC of NR easier; another was due to the overlap of rod-like RC under stretch.

In X-ray diffraction patterns, the two peaks (14.3° and 20.6°) of RRC/NR-3 vulcanizates have almost the same intensity as the pure NR sample, which means that RRC particles had almost no effect on the strain-induced crystallization of NR. However, the results of Figure 6 showed that RRC particles had many obvious influences on the Mullins effect. The causes of the Mullins effect were complex, but it was believed that the filler-filler networks were a major contributing factor [24]. The Mullins effect would be strengthened due to the destruction and reconstruction of filler-filler networks. The enhancement of the Mullins effect under cyclical loading indicated that RRC-RRC networks had taken shape after the strain reached 300%.

Based on the above analysis, it was concluded that the sharp upturn in stress after a 200% strain of RRC/NR composites in Figure 4a was mainly attributed to the formation of the RRC-RRC network instead of the SIC of the NR macromolecular. Because the RRC-filled NR vulcanizates still maintained the same softness as the unfilled ones, as shown in Figure 4b, this sharp upturn in stress by a tensile strain became interesting and valuable. It was well known that the skin of sea cucumbers demonstrated drastic changes in elastic modulus in response to a physical stimulus, and some stimuli-responsive materials had been developed by mimicking this [25,26]. For RRC/NR-3 vulcanizates, it underwent a low modulus (characteristics of soft matter) at a region of small strain, similar to that of unfilled rubber, because the amount of RRC particles was only 3 phr and had been dispersed too well to form a filler network, as shown in the SEM photo in Figure 7a. When a certain strain (≥200%) is reached, the geometric features of the rod-like RC play an important role in forming a filler network quickly, as illustrated in Figure 6b. The rigid RRC-RRC network resulted in a drastic change in stress (or elastic modulus).

## 4. Materials and Methods

### 4.1. Materials

Microcrystalline cellulose was supplied by Shandong Liaocheng Luxi medicinal materials Co., Ltd., Liaocheng, China (particle sizes of 20–90 μm) and NR latex (full ammonia, pH 10–10.5, dry rubber content of 61 wt%) was received from Qingdao Double Butterfly Co., Ltd., Qingdao, China. Sodium hydroxide (NaOH) and analytically pure urea were purchased from Bodie Chemical Engineering Co., Ltd., Tianjin, China. Glacial acetic acid, ethanol, and calcium chloride (CaCl_2_) were provided by the National Pharmaceutical Group Co., Ltd., China. Table 1 gives the compositions of the rubber compounds studied. The term phr represents parts per hundred rubbers in weight. The elastomers were the RRC/NR hybrids with different loadings of cellulose. Other materials, such as zinc oxide, stearic acid, sulfur, and industrial-grade N-tert-butylbenzothiazole-2-sulphenamide (TBBS), were kindly provided by Sentury Tires Co., Ltd., Qingdao, China.

### 4.2. Preparation of RRC/NR Hybrids

**Figure 8:**
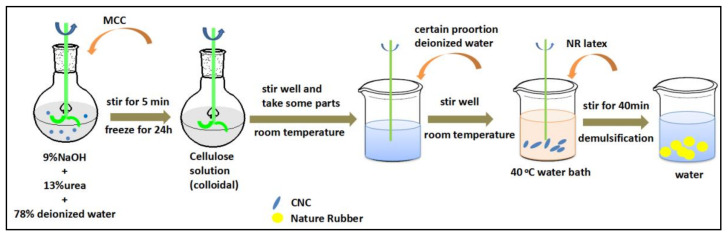
Preparation route for RRC/NR hybrids.

The preparation route for RRC/NR hybrids is shown in Figure 8. First, a 2 wt% cellulose solution was obtained by dissolving a certain amount of cellulose in an NaOH/urea aqueous solution (NaOH:urea:H_2_O = 9:13:78) at −18 °C, as reported [27]. The cellulose solution was diluted with distilled water according to a 1:1 ratio, and then NR latex was dropped into it under vigorous stirring at 40 °C for 30 min. The well-mixed mixture was co-coagulated by adding different coagulants, such as glacial acetic acid, ethanol, 5 wt% CaCl_2_ aqueous, and 5 wt% acetic acid aqueous, respectively. The coagulum was immersed in distilled water at room temperature for 7 days to completely remove NaOH and urea. Finally, the RRC/NR hybrids were obtained by drying the coagulum in an oven at 50 °C for 24 h.

### 4.3. Preparation of the Rubber Compounds

The compounding ingredients listed in Table 1 were weighed out and mixed by a laboratory two-roll mill, and the result was sheets with an approximate thickness of 2 mm. The rubber sheets were vulcanized in a hydraulic press (HS100T-RTMO, Shenzhen Jiaxin Co. Ltd., Shenzhen, China) at 150 °C. The properties of the vulcanizates were measured after standing for 16 h at room temperature.

### 4.4. Characterization

#### 4.4.1. Mechanical Properties

The tensile properties (including tensile strength, modulus at 100% and 300% elongation, and elongation at break) were tested by an electron tensile testing machine (TUM 1445, Zwick, Ulm, Germany) according to the ASTM D412 standards. A uniaxial cyclic mode was carried out to probe the effect of RCC particles on the Mullins effect. The uniaxial cyclic tensile consisted of 3 loading-unloading tests (2 cycles) with maximum strains of 300%. The nominal tensile strain rate of 500 mm/min was applied to these tests. To reduce the uncertainty of the results, at least three specimens were used in each test.

#### 4.4.2. X-ray Diffraction (XRD)

The pure NR and RC/NR samples were both pre-stretched to a 400% strain to make the NR crystallize at room temperature. The XRD spectra of the pre-stretched samples were obtained using a diffractometer (Ultima IV, Rigaku, Tokyo Met., Japan) with a Cu-Kα source at 40 kV and 40 mA.

#### 4.4.3. Scanning Electron Microscopy (SEM)

To observe the morphologies and dispersion states of RRC particles in the NR matrix, SEM photographs of the vulcanizates were taken with a field emission scanning electron microscope (JSM-7500F, Japan Electron Optics Laboratory Co., Ltd., Tokyo Met., Japan). The samples were stretched to failure, and the fracture surfaces were observed. The cross-section was sputter-coated with a thin layer of gold to prevent electrical charging during the examination.

## 5. Conclusions

Cellulose crystals were regenerated in NR latex through a bottom-up self-assembly approach, and their micromorphologies were controlled by adding different co-coagulants to the mixture of cellulose solution and NR latex. The results showed that ideal rod-like cellulose crystals, with a length of about 1.0 μm and a diameter of about 100 nm, were in-situ regenerated and well dispersed in the NR matrix when 5 wt% acetic acid aqueous was used as a coagulant. The rod-like RC exhibited good reinforcement efficiency in NR compounds, especially when its loading reached 3 phr. The RRC particles had little effect on the strain-induced crystallization of NR, but their geometric features played an important role in forming an RRC-RRC filler network when stretched. Once the rigid filler network was formed during the stretching process, a drastic change in stress (or elastic modulus) would occur, exhibiting the potential to be used as stimuli-responsive materials.

## Figures and Tables

**Table 1:** Recipes for the rubber compounds.

Ingredients	Control	RC/NR Composties
	Loading/phr ^1^	Loading/phr
NR	100	0
RC/NR	0	100
stearic acid	2	2
zinc oxide	3	3
sulfur	2.5	2.5
TBBS ^2^	1	1

^1^ parts per hundred rubbers in weight. ^2^ N-tert-butylbenzothiazole-2-sulphenamide.

**Table 2:** Effect of different RC particles on the mechanical properties of RC/NR composites.

Coagulants	Tensile Strength (MPa)	Tear Strength (kN/m)	300% Modulus (Mpa)	Elongation at Break (%)
ethanol	22.6	33.0	2.2	698
5 wt% CaCl_2_	23.0	32.6	2.1	682
glacial acetic acid	24.2	36.1	7.4	563
control	22.4	32.1	2.1	742

## Data Availability

Corresponding authors will provide data if necessary.
